# Predicting Participant Engagement in a Social Media–Delivered Lifestyle Intervention Using Microlevel Conversational Data: Secondary Analysis of Data From a Pilot Randomized Controlled Trial

**DOI:** 10.2196/38068

**Published:** 2022-07-28

**Authors:** Ran Xu, Joseph Divito, Richard Bannor, Matthew Schroeder, Sherry Pagoto

**Affiliations:** 1 Department of Allied Health Sciences Institute for Collaboration in Health, Interventions, and Policy University of Connecticut Storrs, CT United States; 2 Center for Aging Research Indiana University Indianapolis, IN United States

**Keywords:** weight loss, social media intervention, engagement, data science, natural language processing, NLP, social media, lifestyle, machine learning, mobile phone

## Abstract

**Background:**

Social media–delivered lifestyle interventions have shown promising outcomes, often generating modest but significant weight loss. Participant engagement appears to be an important predictor of weight loss outcomes; however, engagement generally declines over time and is highly variable both within and across studies. Research on factors that influence participant engagement remains scant in the context of social media–delivered lifestyle interventions.

**Objective:**

This study aimed to identify predictors of participant engagement from the content generated during a social media–delivered lifestyle intervention, including characteristics of the posts, the conversation that followed the post, and participants’ previous engagement patterns.

**Methods:**

We performed secondary analyses using data from a pilot randomized trial that delivered 2 lifestyle interventions via Facebook. We analyzed 80 participants’ engagement data over a 16-week intervention period and linked them to predictors, including characteristics of the posts, conversations that followed the post, and participants’ previous engagement, using a mixed-effects model. We also performed machine learning–based classification to confirm the importance of the significant predictors previously identified and explore how well these measures can predict whether participants will engage with a specific post.

**Results:**

The probability of participants’ engagement with each post decreased by 0.28% each week (*P*<.001; 95% CI 0.16%-0.4%). The probability of participants engaging with posts generated by interventionists was 6.3% (*P*<.001; 95% CI 5.1%-7.5%) higher than posts generated by other participants. Participants also had a 6.5% (*P*<.001; 95% CI 4.9%-8.1%) and 6.1% (*P*<.001; 95% CI 4.1%-8.1%) higher probability of engaging with posts that directly mentioned weight and goals, respectively, than other types of posts. Participants were 44.8% (*P*<.001; 95% CI 42.8%-46.9%) and 46% (*P*<.001; 95% CI 44.1%-48.0%) more likely to engage with a post when they were replied to by other participants and by interventionists, respectively. A 1 SD decrease in the sentiment of the conversation on a specific post was associated with a 5.4% (*P*<.001; 95% CI 4.9%-5.9%) increase in the probability of participants’ subsequent engagement with the post. Participants’ engagement in previous posts was also a predictor of engagement in subsequent posts (*P*<.001; 95% CI 0.74%-0.79%). Moreover, using a machine learning approach, we confirmed the importance of the predictors previously identified and achieved an accuracy of 90.9% in terms of predicting participants’ engagement using a balanced testing sample with 1600 observations.

**Conclusions:**

Findings revealed several predictors of engagement derived from the content generated by interventionists and other participants. Results have implications for increasing engagement in asynchronous, remotely delivered lifestyle interventions, which could improve outcomes. Our results also point to the potential of data science and natural language processing to analyze microlevel conversational data and identify factors influencing participant engagement. Future studies should validate these results in larger trials.

**Trial Registration:**

ClinicalTrials.gov NCT02656680; https://clinicaltrials.gov/ct2/show/NCT02656680

## Introduction

### Background

Obesity is prevalent in the United States and is a known risk factor for cardiovascular disease, type 2 diabetes [1,2], and cancer. Although lifestyle interventions are effective for weight loss and diabetes prevention [3], they require numerous clinic visits for up to a year, which is burdensome for many people. Technology-delivered lifestyle interventions, by not requiring visits, are less burdensome for participants and have shown promising weight loss outcomes [4]. Some technology-based interventions use popular commercial social media platforms such as Facebook in an effort to meet people where they are [5,6]. Many social media users already use these platforms to discuss their health experiences [7,8]. Community-building features on social media platforms, such as private groups [9,10], make them particularly amenable to delivering group-based lifestyle interventions.

Systematic reviews and meta-analyses show support for the efficacy of social media–delivered lifestyle interventions [4,11]; however, this area of research is still nascent. Participant engagement, defined as posts in the group, replies to posts, “likes,” and votes in polls, appears to be an important predictor of outcomes [12-15]. For example, a study found that every 10 posts by participants corresponded to −0.5% weight loss [16]. Another study found only certain types of engagement predicted weight loss [17]. Interestingly, the degree of participant engagement reported in studies of social media–delivered interventions is highly variable, ranging from an average of once during the entire intervention period to 11 times per week [17-19]. Engagement also generally declines over time in these programs [16]. Our understanding of the factors that influence participant engagement in these interventions is limited. Emerging evidence in the web-based communication literature shows the importance of multilevel factors influencing participant engagement, such as the characteristics of posts (eg, post length and topic and popularity of the poster), characteristics of the conversation thread in response to the post (eg, sentiment and reciprocity behavior), and participant characteristics (eg, motivation and habits) [14,20-27]. However, research is scant in the context of social media–delivered behavioral interventions [28]. Furthermore, characteristics of the conversation thread (ie, other peoples’ replies and comments) preceding each participant’s engagement is often ignored, which could be valuable in terms of providing microlevel contextual data that influences each participant’s decision to engage.

A promising approach to increase our understanding of the factors influencing participants’ engagement in social media–based behavioral interventions is to study the content and interactions generated by the interventionists and participants during the intervention using natural language processing (NLP). Data collected directly from web-based platforms (eg, Facebook) can provide detailed, real-time behavioral information over the course of intervention programs. NLP can handle a large quantity of text, generate reliable qualitative coding [29], be leveraged to derive various real-time microlevel insights concerning the characteristics of the posts (eg, topics) and conversations that followed the posts (eg, sentiment), and understand how they affect participants’ decisions of engagement independently and aggregately. This has potential implications for the design and implementation of future interventions to increase participant engagement, which could lead to more favorable weight loss outcomes.

### Objective

Using data from a 16-week pilot feasibility randomized weight loss trial that delivered lifestyle interventions via Facebook, drawing on multilevel factors influencing participant engagement identified by previous web-based communication literature, we derived various factors from the content generated by participants and interventionists over the course of the intervention, including characteristics of the posts (eg, poster, time, and topic), conversations that followed the post (eg, sentiment and receiving replies), and participants’ previous engagement behaviors, and assessed how well these factors predict participant engagement individually and all together in the context of a social media–delivered lifestyle intervention.

## Methods

### Study Design, Settings, and Participants

In a pilot feasibility randomized trial, we randomized 80 participants who were overweight or obese into 1 of 2 remotely delivered lifestyle interventions. We recruited people interested in losing weight via web-based advertisements at the University of Connecticut on ResearchMatch and in yard sale or neighborhood Facebook groups in 37 states across the United States between June and October of 2019. Inclusion criteria included BMI between 27 and 45 kg/m², smartphone ownership, active Facebook user (ie, comments or posts more than once a week), aged 18 to 65 years, and having daily internet access. Exclusion criteria included pregnancy or planning to become pregnant during the study, bariatric surgery or plans for bariatric surgery during the study, ≥5% weight loss in the past 3 months, pre-existing conditions that preclude physical activity or dietary changes, taking medications affecting weight, incapable of walking one-fourth of a mile unaided without stopping, type 1 or type 2 diabetes, and participation in prior weight loss studies under the principal investigator.

Participants completed an orientation webinar before randomization to learn more about the study, and those still interested in participating were mailed a Wi-Fi scale (FitBit Aria, FitBit Inc) and asked to provide the staff with their log-in information for the scale so that weights could be recorded for the assessments. We randomized 80 participants to the 2 conditions.

### Intervention Conditions

#### Overview

Participants were randomized to either a Facebook group in which new participants were continually enrolled during weeks 1 to 8 (open enrollment) or a Facebook group that included only the original 40 randomized participants (closed enrollment). In the open enrollment condition, 54 additional participants were enrolled between weeks 1 to 8 for a final group size of 94. However, we only included the original 80 randomized participants in this study to ensure all participants had an equal amount of time to engage in all 16 weeks of the intervention.

#### Facebook-Delivered Lifestyle Intervention

Both conditions received the identical 16-week lifestyle intervention based on the Diabetes Prevention Program (DPP) but modified to be delivered in a private Facebook group where twice-daily posts guided participants through the program, which was led by a dietitian (counselor) who was assisted by a student counselor. We adapted the DPP content to be appropriate for a web-based setting, as described elsewhere [30]. We gave each participant an individualized calorie goal that would facilitate a 1 lbs to 2 lbs weight loss weekly and asked them to use MyFitnessPal to track their calories daily. In addition, we asked participants to have the counselor review at least 2 weeks of their MyFitnessPal records, although they could request more as needed. The Facebook group was private such that only those invited by the study team could join the group and view the intervention content. We gave participants diet and exercise goals for the week each Monday and asked them to report progress on their goals in a conversation thread on Sunday and report their weight change for the week in a conversation thread each Friday. In between, intervention posts addressed the learning objectives of each module of the DPP. The dietitian leading the group was instructed to reply to all participant posts and comments that merited a reply and otherwise hit a reaction (eg, like and laugh) button to acknowledge the participants’ comments. Participants were encouraged to post to the group and reply to each other. The details of the intervention, study procedures, and primary study results can be found elsewhere [31].

### Ethics Approval

This pilot feasibility randomized trial was approved by the University of Connecticut Institutional Review Board (H17-215) in October 2017.

### Measures

#### Overview

We included all posts and comments or replies within posts from interventionists and randomized and nonrandomized participants to construct the measures. Posts without text (approximately 6% of the posts were excluded) and polls were excluded, which resulted in 761 posts and 9396 comments or replies across the 2 intervention arms.

The outcome of interest was on the postparticipant pair level; that is, whether each participant had engaged with (ie, commented or replied to) a post in the Facebook group (1 if yes and 0 if no). Comments are in response to the original post, whereas replies are responses to comments made by others on a post. We focused on comments and replies as these activities are active forms of engagement rather than passive types of engagement such as views and reactions (eg, “likes”) and have been shown to positively predict weight loss [16,32,33]. We extracted engagement data from the private Facebook groups using the Grytics app [34]. The Grytics app allowed us to download all the content posted in each Facebook group into Microsoft Excel sheets along with its unique Facebook ID number (post ID, comment ID, and parent comment ID), time stamp, reaction data, and author. Participants were asked to allow the Grytics app to access their Facebook account name so that the content from the group was identified (ie, post or comment author name was included in export).

#### Post Characteristics

The post characteristics described in Textbox 1 were included.

**Post characteristics.**

**Original poster**

- We used a binary variable to indicate whether the focal post was created by the interventionist (1) or participant (0).

**Post length**

- We measured the number of words in each post.

**Content sentiment**

- We measured the average sentiment (text polarity) of each post’s content. Text polarity measures the valence and emotion in the text and ranges on a continuous spectrum from negative (lower value) to positive (higher value). We standardized the measure of sentiment for the analysis. Sentiment analysis was performed using the *sentimentr* package in R (version 3.6.1).

**Topics**

- We used natural language processing to identify the topics that appeared in each post, comment, and reply. The content was preprocessed to remove emojis and non-English characters. Topics were detected using *Top2Vec* in Python 3.10.0, a deep learning–based sentence embedding algorithm that detects topics in the documents. It detected 117 unique topics along with their top words (see Multimedia Appendix 1 for a random sample of 35 topics with their top 8 words) from 10,157 pieces of content (posts, comments, and replies), which were further consolidated and coded under 7 topics based on the top 20 words of each original topic: exercise, diet, weight, MyFitnessPal app use, expressing emotion, sleep, and goals or plans. Here we focused on the topics of each post and created 7 binary variables to represent whether each post involves each of the aforementioned topics. Each post could include multiple topics; for example, a post mentioning a specific dietary goal would be categorized under both diet and goals or plans.

**Time of the post**

- We collected the time (number of days from day 1 of the intervention) and day of the week when the post was created.

#### Reply or Comment Characteristics

We constructed a series of variables representing the characteristics of replies or comments on each post. To reflect the content of conversations before each participant’s engagement, if the participant engaged with the post, we calculated these variables based on all previous comments or replies under the post before their engagement for each unique postparticipant pair; if the participant did not engage with the post, we calculated these measures based on all the comments or replies under the post. The characteristics are described in Textbox 2.

**Reply or comment characteristics.**

**Tags or mentions**

- We created two binary variables to represent whether each participant had been tagged or mentioned by (1) interventionists or (2) other participants in the previous replies or comments within the same post. It is worth mentioning that most tags or mentions in our data were generated automatically by Facebook (eg, when participant A comments or replies to participant B’s content, Facebook automatically generates a tag on B in A’s reply or comment). Thus, most tags or mentions in our data represent reply or comment relationships. In very few instances, interventionists deliberately tagged previously disengaged participants; however, the sample size was too small to test their effects separately.

**Reply or comment content sentiment**

- We measured the average sentiment of all replies or comments for each postparticipant pair. The measure was standardized for the analysis.

#### Participants’ Characteristics

The included participant characteristics were as follows:

- Percentage of previous posts commented or replied: For each post, we calculated the percentage of previous posts each participant has commented or replied to.
- Baseline and sociodemographic characteristics: Although these variables were not the focus of our analysis, we included baseline characteristics for each participant, including treatment condition (open vs closed), baseline weight, BMI, age, race, sex, education, marital status, number of people in the household, and employment status, as covariates in the analyses.

### Statistical Analysis

We focused our analysis on whether each randomized participant (N=80) had engaged with each post, as randomized participants had access to the Facebook group the entire length of the intervention (it should be noted that each post was only available in a particular treatment arm and, thus, can only be seen by 40 randomized participants). To examine what predicts participant engagement with each post, analyses were performed on the postparticipant pair level (ie, whether each participant engaged with each post). This allowed us to construct measures that accurately reflect the content (ie, posts and conversations) before each participant’s engagement. We included all possible engagements (ie, instances where participants engaged and instances where they did not engage) from the 80 randomized participants with each of the 761 posts, which resulted in a final sample of 31,968 observations (participants engaged in 4462 instances and did not engage in 27,506 instances) for our analysis.

**Figure 1:**
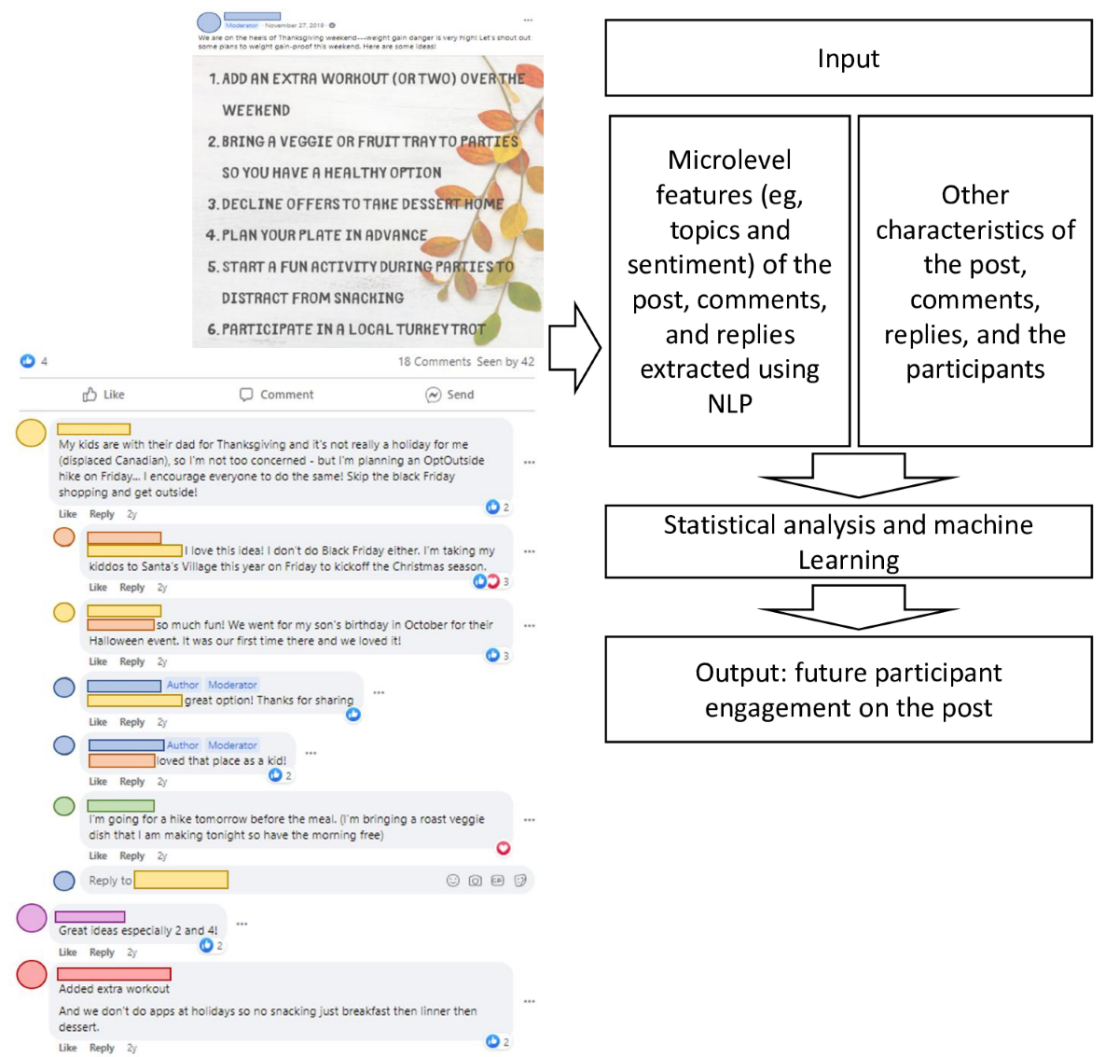
Analysis framework to identify important predictors of participant engagement. Left panel: an example of the intervention post and the comments or replies following it. Right panel: flow chart of the analysis. NLP: natural language processing.

The overall analysis framework is depicted in Figure 1. Data were screened for deviations from assumptions required for the used statistical analyses. We calculated descriptive statistics for the outcome and key independent variables for each treatment condition. To account for the fact that engagement was nested within each post, we performed a mixed-effects regression with postlevel random effects using participants’ engagement as the primary outcome, along with all aforementioned key predictors (ie, characteristics of the post, reply or comment, and the participants’ previous engagement behavior) as independent variables, with participants’ baseline and sociodemographic characteristics as covariates. We also included participant-level fixed effects as an alternative specification to account for possible omitted variable bias. As a robustness check, we also conducted a mixed-effects logistic regression with the same variables (Multimedia Appendix 1). To identify the important predictors of participant engagement, we reported the coefficient, 95% CI, and the associated *P* value for each predictor. All analyses were performed in STATA standard edition (version 16).

Although regression analyses are useful to identify the statistical significance of linear relationships, some of the relationships might be much more complex (eg, nonlinear or moderated by other variables). To confirm the importance of significant predictors that we previously identified and to investigate how well these variables as a whole can predict participants’ engagement with a particular post, we included all aforementioned predictors in machine learning algorithms, including gradient boosting machines, deep learning models, and an ensemble of them [35,36], and examined the performance of these models by calculating key metrics, including (1) area under the curve (ranging from 0.5 to 1, with 1 being the best) using a 5-fold cross-validation, (2) variable importance across different models, and (3) out-of-sample prediction accuracies on a balanced sample with 1600 observations (800 random observations with engagement and 800 observations without). In the machine learning model, variable importance was determined by calculating the relative influence of each variable: in the tree-based model, it was calculated based on whether that variable was selected or included during the tree-building process and how much it improves the model fit. In other non–tree-based models, it was calculated as the magnitude of the weight or coefficient connecting a specific input or variable to the output [37]. We also evaluated the variable importance using an alternative approach called Shapley Additive Explanations contribution in one of the top performing models, which measures how much the average model prediction would change with and without a specific feature or variable [38], as shown in Multimedia Appendix 1. All the analyses were performed using the *h2o* package [39] in R (R Foundation for Statistical Computing; version 3.6.1).

## Results

Table 1 presents the sociodemographic characteristics and key measures of engagement for the participants (N=80). The mean age of participants was 40.2 (SD 11.2) years with a mean baseline BMI of 34.4 (SD 5.0) kg/m^2^. Participants were predominantly female (68/80, 85%) and mostly White (72/80, 90%) White, with most (58/80, 73%) reporting completing college or graduate school. The baseline characteristics of the participants in the 2 treatment conditions were similar, and we did not observe significant differences in these variables between the groups. Over the 16-week intervention, participants in the closed group commented or replied to 9.9% (37/374; SD 10.4%) of the posts on average, whereas participants in the open group commented or replied to 8.8% (34/387; SD 9.7%) of the posts on average.

Table 2 presents the key characteristics of the posts and comments or replies generated over the 16-week intervention across the 2 treatment arms. Post length was 33.4 words on average (SD 23.81) and 57.3% (436/761) of the posts were created by the interventionists. It should be noted participants in the 2 groups were exposed to identical program posts (whereas the number of self-generated posts by interventionists could be different). Topic modeling results showed that diet was the most popular topic across all posts (310/761, 40.7%), followed by exercise (163/761, 21.4%), goal or plan (152/761, 19.9%), and weight (138/761, 18.1%). We did not observe significant differences in post or comment or reply characteristics across groups, except that the percentage of replies or comments directed at randomized participants was significantly higher in the closed group than the open group (*P*<.001), possibly because there were 54 nonrandomized participants in the open group.

**Table 1:** Participant characteristics (N=80).

Participant characteristics		Closed enrollment (n=40)	Open enrollment (n=40)
Age (years), mean (SD)		40.4 (11.8)	40.0 (10.6)
Female, n (%)		34 (85)	34 (85)
Baseline BMI (kg/m^2^), mean (SD)		34.8 (5.4)	34.0 (4.6)
Hispanic or Latino, n (%)		3 (8)	1 (3)
**Race, n (%)**			
	White	36 (90)	36 (90)
	Black or African American	3 (8)	3 (8)
	Asian	0 (0)	0 (0)
	Native Hawaiian or other Pacific Islander	0 (0)	0 (0)
	American Indian or Alaska Native	0 (0)	0 (0)
	Multiethnic	0 (0)	1 (3)
	Unknown	1 (3)	0 (0)
**Marital status, n (%)**			
	Married or living with partner but not married	29 (73)	30 (75)
	Single	8 (20)	6 (15)
	Widowed, divorced, or separated	3 (8)	4 (10)
**Education, n (%)**			
	Less than high school, high school degree, GED^a^, equivalent	1 (3)	2 (5)
	Trade, technical, some college, associates	8 (20)	11 (28)
	Bachelor’s degree or some graduate school	21 (53)	17 (43)
	Graduate degree	10 (25)	10 (25)
**Employment status, n (%)**			
	Employed full-time	28 (70)	27 (68)
	Employed part-time	7 (18)	4 (10)
	Student	2 (5)	2 (5)
	Unemployed, retired, disabled, or homemaker	3 (8)	6 (15)

^a^GED: General Educational Development.

**Table 2:** Post and reply or comment characteristics over the 16-week intervention.

Post characteristics		Closed enrollment (n=374)	Open enrollment (n=387)
Content sentiment, mean (SD)		0.134 (0.197)	0.133 (0.195)
Number of words, mean (SD)		33.78 (24.63)	33.28 (23.12)
Created by interventionists, n (%)		225 (60.2)	211 (54.5)
**Topic, n (%)**			
	Exercise	80 (21.4)	83 (21.4)
	Diet	158 (42.2)	152 (39.3)
	Weight	64 (17.1)	74 (19.1)
	MyFitnessPal app	61 (16.3)	67 (17.3)
	Expressing emotion	28 (7.5)	28 (7.2)
	Sleep	6 (1.6)	5 (1.3)
	Goals or plans	80 (21.4)	72 (18.6)
**Reply or comment characteristics^a^**			
	Content sentiment, mean (SD)	0.171 (0.255)	0.156 (0.234)
	Participant’s reply to other participants, n (%)	750 (23.8)	803 (12.9)
	Interventionist reply to a participant, n (%)	1018 (32.3)	1195 (19.1)

^a^Closed enrollment n=3152 and open enrollment n=6244.

Table 3 shows the results from mixed-effects regression models on how well each variable predicted participants’ engagement with a specific post. Regarding post characteristics, we found that the overall probability of participants’ engagement with each post decreased by 0.04% each day (*P*<.001; 95% CI 0.02%-0.06%). Participants had a 6.3% (*P*<.001; 95% CI 5.1%-7.5%) higher probability of engaging with posts generated by the interventionists than with posts created by other participants. Post length also mattered—one additional word in a post’s content was associated with a 0.05% (*P*<.001; 95% CI 0.03%-0.08%) increase in participants’ probability of engagement. Participants also had a 6.5% (*P*<.001; 95% CI 4.9%-8.1%) and 6.1% (*P*<.001; 95% CI 4.1%-8.1%) higher probability of engaging with posts if the post content was related to weight and goals or plans, respectively. Regarding reply or comment characteristics, participants were 44.8% (*P*<.001; 95% CI 42.8%-46.9%) more likely to engage with a post when they received replies from other participants in the conversation or 46% (*P*<.001; 95% CI 44.1%-48.0%) more likely to engage if they received replies by interventionists. A 1 SD decrease in the sentiment in the previous replies or comments was associated with a 5.4% (*P*<.001; 95% CI 4.9%-5.9%) increase in the probability of participant engagement. Participants’ engagement in previous posts was a strong predictor of future engagement—a 1% increase in participants’ previous engagement was associated with a 0.76% (*P*<.001; 95% CI 0.74%-0.79%) increase in their probability to engage with a subsequent post. Robustness analyses showed that these results were largely consistent with the results from (1) multivariate linear regression results and mixed-effects regression with participant-level fixed effects and (2) mixed-effects logistic regression with or without postlevel random effects and participant fixed effects. Details of the results from these additional regression analyses can be found in Multimedia Appendix 1.

**Figure 2:**
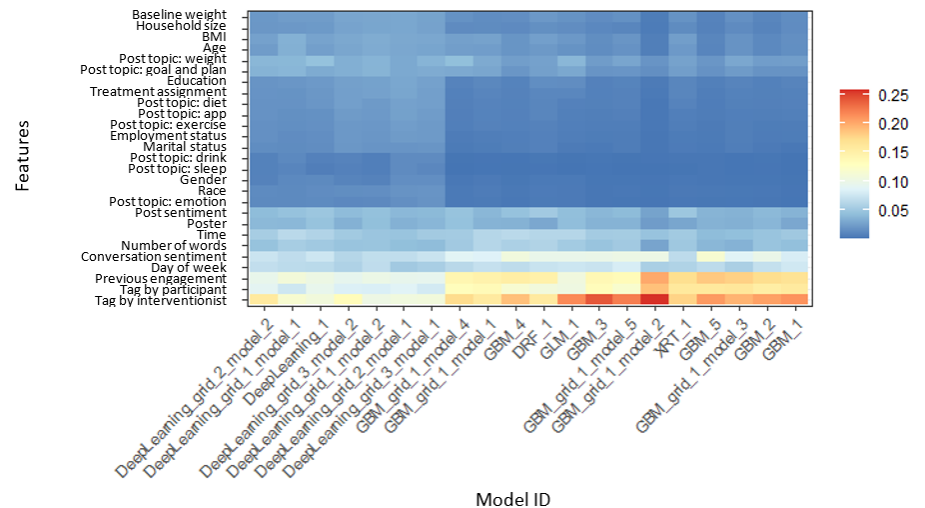
Variable importance of predicting participant engagement across 20 machine learning models. The x-axis shows different model names, and variables from top to bottom on the y-axis are baseline weight, number of people in the household, baseline BMI, age, post topic weight, post topic goal or plan, education, treatment assignment, post topic diet, post topic MyFitnessPal app, post topic exercise, employment status, marital status, post topic drink, post topic sleep, gender, race, post topic expressing emotion, post sentiment, whether the post is created by interventionists, day of the intervention when the post is created, word count of the post, replies or comments sentiment, day of the week when the post is created, percentage of previous posts engaged, and whether replied by other participants or by interventionists.

To confirm the importance of previously identified predictors and test how well the aforementioned variables can predict the probability of a participant engaging with a post, we performed a variety of machine learning–based classification algorithms with all the aforementioned predictors as the input and participant engagement as the outcome. Of the 32 models we tested, the ensemble approach of gradient boosting machine learning–based and deep learning–based classification algorithms performed the best, with an average area under the curve of 0.963 using 5-fold cross-validation (see more results in Multimedia Appendix 1). Figure 2 shows the variable importance across 20 machine learning models (excluding ensemble models) we tested, with those indicated by yellow and red being more important variables. The results show that receiving a reply from other participants and interventionists, percentage of previous posts participants had engaged in, average sentiment in previous replies or comments, time of the post, and day of the week were the most important variables across models, which were consistent with the results from regression analyses. Finally, we performed out-of-sample predictions on a balanced sample with 1600 observations (800 observations with engagement and 800 observations without) and achieved 90.9% accuracy and 0.908 F_1_ score at maximum.

**Table 3:** Mixed-effects regression results predicting participants’ engagement (N=31,968)^a^.

Mixed-effects regression			Values, mean (SD; range)	Coefficient (95% CI)	*P* value
Outcome: participants’ engagement			0.140 (0.347; 0 to 1)	—^b^	—
**Post characteristics**					
	Created by interventionists		0.583 (0.493; 0 to 1)	0.0627 (0.0507 to 0.0746)	<.001
	Number of words		33.44 (23.80; 1 to 107)	0.0005 (0.0003 to 0.0008)	<.001
	Content sentiment (standardized)		0 (1; −3.67 to 4.74)	−.0042 (−0.097 to 0.0012)	.13
	**Topics**				
		Exercise	0.212 (0.409; 0 to 1)	0.0096 (−0.0075 to 0.0266)	.27
		Diet	0.389 (0.487; 0 to 1)	−0.0085 (−0.0249 to 0.0078)	.31
		Weight	0.191 (0.392; 0 to 1)	0.0654 (0.0494 to 0.0814)	<.001
		MyFitnessPal app	0.163 (0.369; 0 to 1)	−0.0377 (−0.0534 to −0.0219)	<.001
		Expressing emotion	0.071 (0.257; 0 to 1)	0.0083 (−0.01558 to 0.0321)	.50
		Sleep	0.0141 (0.117; 0 to 1)	−0.0587 (−0.1070 to −0.0103)	.02
		Goals or plans	0.209 (0.407; 0 to 1)	0.0612 (0.0414 to 0.0811)	<.001
	Time of the post		46.61 (32.94; 1 to 112)	−0.0004 (−0.0006 to −0.0002)	<.001
**Reply or comment characteristics**					
	Content sentiment (standardized)		0 (1; −7.84 to 5.91)	−0.0539 (−0.0589 to −0.0488)	<.001
	Replied by other participants		0.026 (0.161; 0 to 1)	0.4484 (0.4279 to 0.4690)	<.001
	Replied by interventionists		0.029 (0.167; 0 to 1)	0.4604 (0.4409 to 0.4798)	<.001
**Participants characteristics**					
	Percentage previous posts commented or replied		13.16 (13.68; 0 to 100)	0.0076 (0.0074 to 0.0079)	<.001

^a^The model included postlevel random effects and also controlled for day of the week when the post was created and other baseline and sociodemographic characteristics of the participants, including treatment assignment, race, marital status, education, employment, number of people in the household, age, gender, baseline BMI, and weight.

^b^Not available.

## Discussion

### Principal Findings

In this study, we conducted secondary analyses using data from a 2-arm pilot feasibility randomized controlled trial that delivered lifestyle interventions via Facebook. We analyzed commenting or replying behavior from 80 participants in response to each of the 761 posts generated by counselors and participants over the 16-week intervention period and linked them to predictors, including post characteristics (eg, time, post length, and post topic), conversation characteristics (eg, sentiment of the conversation and participants being replied to), and participant characteristics (eg, sociodemographics and previous commenting or replying behavior). Our findings suggest that although participants’ comments or replies decreased over time, important characteristics of the post, the conversation attached to that post, and the participants’ engagement patterns predicted whether a participant engaged with a specific post. For example, we found that participants who engaged more with prior posts were more likely to engage with future posts. Posts that were longer (with the maximum number of words not exceeding 107), were created by interventionists, or had content related to weight (eg, weigh-in posts) and goal setting are more likely to attract engagement. The latter is consistent with the design of the intervention—participants were asked to set diet and exercise goals for the week each Monday, report their progress on their goals on Sunday, and report their weight change for the week each Friday. This is also encouraging because goal setting [40] and frequent self-weighing [41] are key behavioral weight loss strategies. Furthermore, posts with replies or comments that directly mentioned the focal participant were much more likely to attract subsequent replies from that participant. Moreover, posts with replies or comments that contained negative sentiments were more likely to attract subsequent comments. This is likely because participants who share struggles, problems, and challenges are naturally more negative in sentiment (see Multimedia Appendix 1 for examples of replies or comments with negative sentiments), and such content often attracts support and brainstorming from other participants who may feel called to help when others are struggling. These results were robust to multiple alternative specifications. Machine learning results also show that together, these characteristics can predict participant engagement with a high accuracy of 90.9%.

### Implications

In this study, we demonstrate the potential of using NLP tools to analyze microlevel conversational data and identify factors influencing participants’ commenting or replying behavior in a social media–delivered weight loss intervention. Our findings shed light on some important microlevel characteristics of the participants, posts, and conversations, which can shape participants’ experiences during the intervention and predict their future engagement. These results have implications for the design and implementation of social media–delivered behavioral interventions in ways that maximize participant engagement. We previously reported a strong association between participant engagement and weight loss [31], which suggests that engagement-enhancing strategies could lead to more favorable outcomes. For example, enhancing engagement early on may help with sustained engagement. Furthermore, we found that receiving replies appears to stimulate further engagement from the participant. This may also be a function of whether a participant shares something substantive about themselves in a comment. For example, if a participant’s comment is “thanks!” the interventionist may just hit the “like” button; however, if a participant’s comment is a question or sharing of a goal, the interventionist and other participants are more likely to reply to continue that conversation. Further research should explore the type of comments (eg, questions, sharing a problem, and setting a goal) that are most likely to elicit replies from others. Program content should be designed in ways that nudge participants to post more often and interact more with each other (eg, use open-ended questions and encourage peer-to-peer support). Although in this study we found that participants were more likely to respond to interventionists, greater peer-to-peer engagement could also facilitate stronger group cohesion, thereby further enhancing participant engagement. Posts in which participants share struggles, problems, and challenges they have encountered during the weight loss process may draw more participants into the conversation, which may generate richer brainstorming and social support, both of which could also enhance group cohesion. These implications could be applicable not only to social media–delivered weight loss interventions but also to other digital health interventions more generally.

### Comparison With Prior Work

Although many studies have tested social media–delivered weight loss interventions or emphasized the importance of participant engagement in web-based communities [12-15,42], only a handful of studies have identified the factors that can influence participant engagement in digital health interventions [43,44], and most of them focused on macrolevel factors such as post type and participants’ characteristics (eg, age and gender) [9,14]. Few studies have examined microlevel factors such as conversation dynamics. Several studies recognized this limitation and called for more research to identify all relevant factors that can predict participant engagement [28,43,45,46]. This study contributes to this line of research in two ways: (1) we demonstrate how content generated by interventionists and participants during the course of a digital intervention can be leveraged and combined with data science and NLP tools to identify microlevel predictors of participant engagement, and (2) we have identified many microlevel factors that influence participant engagement, which, to the best of our knowledge, have not been studied in previous social media–based behavioral interventions. This has practical implications for future intervention designs that can maximize participant engagement.

Similar to previous studies, we found that participant engagement is highly variable [17-19], and it generally declines over time [16,47]. Although many factors identified in this study have not been studied in the social media–based behavioral intervention context, our findings are consistent with psychological and sociological theories, as well as several empirical research on web-based communication. For example, our finding that posts created by interventionists are more popular is consistent with other studies on web-based communities, showing that important users or those with designated roles are more likely to draw responses from other users [26], possibly because of preferential attachment [48]. Our finding that replies or comments with negative sentiments draw more engagement implies that participants are more likely to reply or comment when they see others sharing their struggles and challenges. This could be possibly explained by social support processes, which have substantial empirical support across various web-based settings [22-25,49]. Finally, the importance of being replied to by interventionists and other participants can be explained by the preference for reciprocity [50], which has been found to be an important driver for communication in many other web-based settings [20,21].

### Limitations and Future Work

This study has several limitations that point to avenues for future research. First, our sample size was small (80 participants; 10,157 total posts, comments, and replies) and our participants were predominantly White (72/80, 90%) and female (68/80, 85%). This limits the generalizability of our results, following a long-standing pattern in weight loss studies of difficulty recruiting male participants [51]. Similarly, given that 96% (77/80) of our participants reported attaining a college degree or advanced degree, we cannot generalize our results to individuals with lower levels of education. Future studies should devise recruitment strategies that attract more male participants and participants with low levels of education to further explore the individual heterogeneity across people from different backgrounds. Second, this study did not fully tease out all possible confounding factors and thus cannot establish causality. For instance, participants who are more successful in losing weight might also be more likely to comment simply because they are paying more attention to the group and have more to say as they are applying the knowledge and strategies they are learning. Future studies should include larger trials, surveys with more longitudinal measures (eg, physical activity and diet tracking, mental health, and other behaviors), and qualitative studies to establish the possible bidirectional and causal relationships between engagement and these factors. Third, we focused on replies and comments and did not explore other types of engagement such as reactions and views of posts and comments. Although comments and replies have been considered more substantive than other engagements (eg, likes and “lurking”), other engagements potentially comprise a substantial proportion of social media use and thus warrant careful consideration in future studies [33]. Similarly, although we included participants’ posts in our analysis, we did not include posts only with images or videos or investigate the factors influencing participants’ decisions to create posts, which is another important form of engagement. In addition, although we included tags or mentions relationships in our study, most tags were automatically generated by Facebook during replies or comments. Future studies should consider whether deliberate tagging can nudge disengaged participants to re-engage with the program. Fourth, we assumed all participants had an opportunity to engage with every post in the group, and we considered all replies or comments when constructing the predictors for participants who did not engage with a certain post, which might not necessarily be the case if the participant did not see the post or the previous comments or replies. Future studies could take additional information into account, such as what participants viewed and the time a participant spends on Facebook. This will allow researchers to construct more refined measures to reflect the condition under which participants make a decision of whether to engage. Finally, although we observed that longer posts are more likely to draw engagement from participants, it should be noted the posts in this intervention were generally short by design (mean 33.4, maximum 107 words). Social media marketing reports reveal that Facebook posts that have <50 characters receive the highest level of engagement relative to longer posts [52]. A/B testing of a wide range of post lengths and different types of posts (eg, goal setting vs problem sharing) is needed to determine the ideal length of posts to maximize engagement in behavioral interventions.

### Conclusions

In this study, we performed secondary analyses using data from a pilot feasibility randomized weight loss trial that delivered a lifestyle intervention via Facebook and linked participants’ engagement with several important predictors, including characteristics of the posts, replies or comments, and participants. Our results point to the potential of using data science and NLP tools to analyze microlevel behavioral or conversational data and identify factors influencing participants’ engagement during the social media weight loss intervention, which have implications for the design and implementation of future interventions that could lead to more favorable weight loss outcomes. Future studies are warranted to validate our results and further explore these relationships in similar and larger trials.

## Appendix

Multimedia Appendix 1 Supplementary analyses and results.

## Abbreviations

DPP: Diabetes Prevention Program
NLP: natural language processing
