# Supplementary material for: Predicting Participant Engagement in a Social Media–Delivered Lifestyle Intervention Using Microlevel Conversational Data: Secondary Analysis of Data From a Pilot Randomized Controlled Trial
Source: JMIR Form Res. 2022 Jul 28;6(7):e38068. doi: 10.2196/38068 (PMC9377444; doi:10.2196/38068)
Supplement: Multimedia Appendix 1 [file formative_v6i7e38068_app1.docx]

**Multimedia Appendix 1. Supplementary analyses and results.**

In this appendix we included (1) full regression results of the main analysis including those from linear regression, mixed effects regression, and mixed effects regression with participant level fixed effects; (2) robustness analysis results using logistic regression, mixed effects logistic regression, and mixed-effects logistic regression with participant level fixed effects using the same variables as the main analysis; (3) variable importance in terms of SHapley Additive exPlanations (SHAP) contribution in one of the top performing machine learning models; (4) Receiver operating characteristics (ROC) curve from the best performing machine learning model predicting participant’s engagement on an independent balanced sample with 1,600 observations; (5) A random sample of replies/comments with negative sentiment. (6) A random sample of 40 topics along with their top 8 words identified by topic modeling.

Table S1. Results from various regression specifications to predict participant’s engagement

|  | | (1) | | (2) | | (3) | |
| --- | --- | --- | --- | --- | --- | --- | --- |
| Outcome: participant engagement | | Linear regression | | Mixed effects regression | | Mixed effects regression with participant fixed effects | |
|  | |  | |  | |  | |
| Posted by interventionist | | 0.0823*** | | 0.0627*** | | 0.0681*** | |
|  | | (0.00352) | | (0.00609) | | (0.00518) | |
| # of words | | 0.000499*** | | 0.000546*** | | 0.000550*** | |
|  | | (7.31e-05) | | (0.000127) | | (0.000108) | |
| Content sentiment | | -0.0315*** | | -0.0219 | | -0.0217 | |
|  | | (0.00834) | | (0.0145) | | (0.0123) | |
| Topic: exercise | | 0.0139** | | 0.00955 | | 0.000213 | |
|  | | (0.00495) | | (0.00871) | | (0.00740) | |
| Topic: diet | | 0.00192 | | -0.00854 | | -0.0130 | |
|  | | (0.00477) | | (0.00837) | | (0.00711) | |
| Topic: weight | | 0.0698*** | | 0.0654*** | | 0.0621*** | |
|  | | (0.00462) | | (0.00817) | | (0.00693) | |
| Topic: app | | -0.0328*** | | -0.0377*** | | -0.0270*** | |
|  | | (0.00463) | | (0.00804) | | (0.00684) | |
| Topic: emotion | | 0.0102 | | 0.00830 | | -0.00138 | |
|  | | (0.00702) | | (0.0122) | | (0.0104) | |
| Topic: sleep | | -0.0375** | | -0.0587* | | -0.0574** | |
|  | | (0.0142) | | (0.0247) | | (0.0210) | |
| Topic: goal_plan | | 0.0664*** | | 0.0613*** | | 0.0546*** | |
|  | | (0.00573) | | (0.0101) | | (0.00859) | |
| Reply/comment sentiment | | -0.138*** | | -0.344*** | | -0.255*** | |
|  | | (0.0102) | | (0.0165) | | (0.0144) | |
| Replied by other participants | | 0.518*** | | 0.448*** | | 0.446*** | |
|  | | (0.0106) | | (0.0105) | | (0.0105) | |
| Replied by interventionists | | 0.524*** | | 0.460*** | | 0.454*** | |
|  | | (0.0101) | | (0.00992) | | (0.00991) | |
| Day of the week (Tuesday) | | -0.0304*** | | -0.0202 | | -0.0223* | |
|  | | (0.00616) | | (0.0108) | | (0.00916) | |
| Day of the week (Wednesday) | | -0.0307*** | | -0.0216* | | -0.0276** | |
|  | | (0.00597) | | (0.0104) | | (0.00886) | |
| Day of the week (Thursday) | | -0.0331*** | | -0.0279** | | -0.0323*** | |
|  | | (0.00584) | | (0.0102) | | (0.00868) | |
| Day of the week (Friday) | | 0.0328*** | | 0.0302** | | 0.0234** | |
|  | | (0.00594) | | (0.0105) | | (0.00888) | |
| Day of the week (Saturday) | | -0.0350*** | | -0.0259* | | -0.0363*** | |
|  | | (0.00594) | | (0.0103) | | (0.00879) | |
| Day of the week (Sunday) | | -0.0298*** | | -0.0213* | | -0.0332*** | |
|  | | (0.00579) | | (0.0101) | | (0.00860) | |
| Day of the intervention | | -0.000594*** | | -0.000404*** | | -0.00109*** | |
|  | | (5.07e-05) | | (8.68e-05) | | (7.32e-05) | |
| % previous posts engaged | | 0.00689*** | | 0.00764*** | |  | |
|  | | (0.000132) | | (0.000134) | |  | |
| Constant | | -0.00565 | | 0.0403 | | 0.0606*** | |
|  | | (0.0199) | | (0.0229) | | (0.0170) | |
| Baseline and socio-demographic characteristics | | X | | X | |  | |
|  | |  | |  | |  | |
| Observations | | 31,968 | | 31,968 | | 31,968 | |
| R-squared | | 0.374 | | 0.362 | | 0.388 | |
| Number of unique posts | | 761 | | 761 | | 761 | |

Standard errors in parentheses

*** p<0.001, ** p<0.01, * p<0.05

Table S2. Results from various logistic regression specifications to predict participant’s engagement

|  | | (1) | | (2) | | (4) | |
| --- | --- | --- | --- | --- | --- | --- | --- |
| Outcome: participant engagement | | Logistic Regression | | Mixed effects logistic regression | | Mixed effects logistic regression with participant fixed effects | |
|  | |  | |  | |  | |
| Posted by interventionist | | 1.319*** | | 1.098*** | | 1.125*** | |
|  | | (0.0564) | | (0.168) | | (0.154) | |
| # of words | | 0.00813*** | | 0.0136*** | | 0.0134*** | |
|  | | (0.00109) | | (0.00351) | | (0.00323) | |
| Content sentiment | | -0.539*** | | -0.876* | | -0.705 | |
|  | | (0.131) | | (0.412) | | (0.378) | |
| Topic: exercise | | 0.139 | | -0.0489 | | -0.141 | |
|  | | (0.0725) | | (0.242) | | (0.222) | |
| Topic: diet | | -0.0204 | | -0.415 | | -0.473* | |
|  | | (0.0698) | | (0.233) | | (0.213) | |
| Topic: weight | | 0.772*** | | 0.732*** | | 0.749*** | |
|  | | (0.0575) | | (0.218) | | (0.200) | |
| Topic: app | | -0.674*** | | -0.870*** | | -0.637** | |
|  | | (0.0808) | | (0.226) | | (0.207) | |
| Topic: emotion | | 0.0164 | | -0.0140 | | -0.130 | |
|  | | (0.104) | | (0.336) | | (0.309) | |
| Topic: sleep | | -0.582* | | -1.636* | | -1.507* | |
|  | | (0.232) | | (0.680) | | (0.624) | |
| Topic: goal_plan | | 0.627*** | | 0.573* | | 0.512* | |
|  | | (0.0746) | | (0.274) | | (0.251) | |
| Reply/comment sentiment | | -2.315*** | | -8.118*** | | -7.061*** | |
|  | | (0.166) | | (0.452) | | (0.430) | |
| Replied by other participants | | 8.812*** | | 9.264*** | | 8.988*** | |
|  | | (1.005) | | (1.056) | | (1.036) | |
| Replied by interventionists | | 5.875*** | | 6.059*** | | 6.367*** | |
|  | | (0.335) | | (0.382) | | (0.440) | |
| Day of the week (Tuesday) | | -0.391*** | | -0.0660 | | -0.121 | |
|  | | (0.0870) | | (0.294) | | (0.269) | |
| Day of the week (Wednesday) | | -0.331*** | | 0.0189 | | -0.134 | |
|  | | (0.0826) | | (0.283) | | (0.259) | |
| Day of the week (Thursday) | | -0.342*** | | -0.107 | | -0.222 | |
|  | | (0.0800) | | (0.276) | | (0.253) | |
| Day of the week (Friday) | | 0.454*** | | 0.485 | | 0.348 | |
|  | | (0.0765) | | (0.284) | | (0.259) | |
| Day of the week (Saturday) | | -0.365*** | | -0.0580 | | -0.268 | |
|  | | (0.0849) | | (0.283) | | (0.259) | |
| Day of the week (Sunday) | | -0.259** | | -0.0438 | | -0.284 | |
|  | | (0.0811) | | (0.277) | | (0.254) | |
| Day of the intervention | | -0.00867*** | | -0.00560* | | -0.0163*** | |
|  | | (0.000735) | | (0.00238) | | (0.00219) | |
| % previous posts engaged | | 0.0592*** | | 0.0897*** | |  | |
|  | | (0.00159) | | (0.00222) | |  | |
| Constant | | -4.597*** | | -3.956*** | | -4.593*** | |
|  | | (0.306) | | (0.520) | | (0.548) | |
| Baseline and socio-demographic characteristics | | X | | X | |  | |
|  | |  | |  | |  | |
| Observations | | 31,968 | | 31,968 | | 31,218 | |
| Number of unique posts | | 761 | | 761 | | 761 | |

Standard errors in parentheses

*** p<0.001, ** p<0.01, * p<0.05


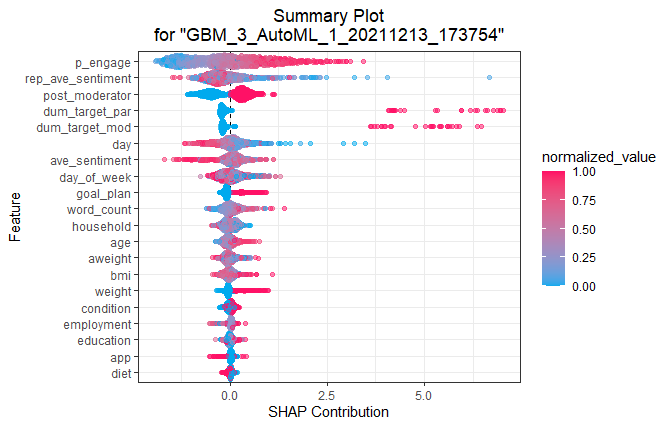


Figure S1. Variable importance in terms of SHAP contribution in one of the top performing machine learning models (a gradient boosting machine). Variables on top are the more important ones (i.e., previous engagement, conversation sentiment, whether the poster is the moderator, replied by participants and replied by the moderator).


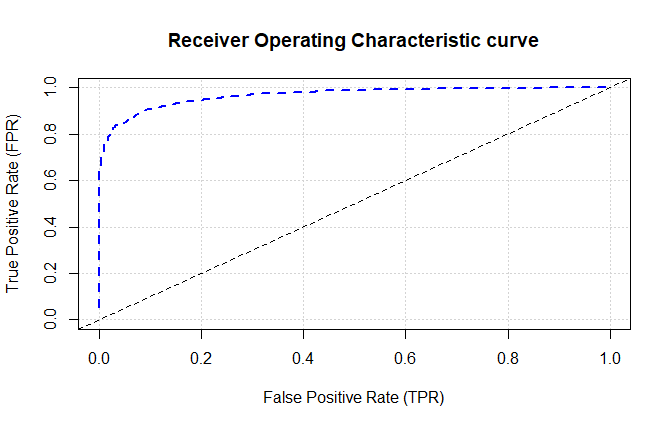


Figure S2. Receiver operating characteristic curve of the best performing machine learning model to predict participant engagement in an independent balanced sample with 1,600 observations

Table S3. Some examples of replies/comments with negative sentiment

| Reply/comment sentiment | Reply/comment text |
| --- | --- |
| -0.44274 | You are welcome to but I think I'd be lying to myself if I pretended that I don't know the problem...too many unhealthy snacks and sweets. |
| -0.51962 | Depression for sure. |
| -0.51188 | Naps always wind up making me feel more tired (I suspect because I'm not able to get deep enough sleep while napping...) - I envy people who can nap and feel rejuvenated |
| -1.12268 | Anger, and consuming too much caffeine. |
| -0.14434 | The feeling of failure when you don't make your goal. |
| -0.4773 | Stress, happy, EXHAUSTED more than any other though. |
| -0.39434 | typically I can manage it but I struggle at times to not eat it more than once a day. |
| -0.34739 | more exercise and getting to bed earlier. And logging.... I'm afraid I've really failed at that one so far |
| -0.15135 | I try not to weigh daily when I'm retaining water...because it's rather depressing when you're up four pounds overnight for hormonal reasons. |
| -0.19285 | Its been a hell of a week. Stress is getting the best of me this month. |

Table S4. A random sample of 40 topics with their top 8 words identified by topic modeling

| Topic | topword1 | topword2 | topword3 | topword4 | topword5 | topword6 | topword7 | topword8 |
| --- | --- | --- | --- | --- | --- | --- | --- | --- |
| 13 | favorite | proud | weekly | biggest | welcome | amazing | best | excellent |
| 25 | goals | goal | motivation | motivated | healthy | focused | progress | focus |
| 19 | sleep | night | bed | routine | stress | exercise | morning | habit |
| 78 | pace | fast | run | running | treadmill | walking | exercising | excellent |
| 17 | veggies | meals | vegetables | meal | recipes | recipe | foods | dinner |
| 88 | welcome | group | awesome | happy | Glad | proud | hi | fun |
| 50 | nice | great | awesome | amazing | good | excellent | wow | super |
| 26 | thanks | awesome | wow | nice | amazing | excellent | thank | great |
| 21 | exercise | exercising | workouts | workout | activity | cardio | fitness | routine |
| 54 | mfp | myfitnesspal | fitbit | calorie | calories | carbs | servings | diet |
| 24 | thoughts | favorite | how | ways | ideas | suggestions | what | things |
| 60 | good | nice | enjoy | great | excellent | awesome | okay | amazing |
| 100 | love | nice | awesome | sweet | Wow | amazing | great | fun |
| 28 | chocolate | snack | sweets | dessert | snacks | fruit | fruits | candy |
| 87 | stress | stressful | exercise | fun | losing | hungry | motivation | motivated |
| 84 | friday | weigh | weight | monday | pounds | sunday | weekly | lbs |
| 42 | goals | monday | weekly | week | sunday | motivation | workouts | weekends |
| 89 | nice | recipe | looks | pretty | awesome | amazing | wow | excellent |
| 104 | dinner | pounds | lunch | candy | Eat | nice | days | happy |
| 4 | walking | hike | walk | walked | treadmill | exercise | exercising | running |
| 99 | fruits | protein | veggies | diet | fiber | fruit | carbs | healthier |
| 18 | candy | sweets | halloween | snacks | snack | chocolate | sugar | thanksgiving |
| 11 | exercise | workout | workouts | exercising | work | motivation | fitness | treadmill |
| 47 | gym | workout | exercising | workouts | fitness | treadmill | exercise | cardio |
| 6 | myfitnesspal | mfp | calorie | calories | fitbit | servings | carbs | diet |
| 73 | cardio | weight | myfitnesspal | fat | exercising | mfp | workouts | workout |
| 68 | recipe | recipes | servings | cooking | meals | dessert | meal | make |
| 65 | weigh | scale | weight | friday | pounds | sunday | monday | lbs |
| 16 | workouts | cardio | workout | exercising | exercise | treadmill | fitness | gym |
| 97 | awesome | welcome | wow | nice | amazing | happy | great | excellent |
| 83 | welcome | hi | awesome | great | amazing | enjoy | fun | wow |
| 10 | veggies | vegetables | rice | servings | chicken | meal | meals | carbs |
| 74 | thanks | thank | great | awesome | okay | ok | wow | please |
| 2 | eating | snacks | snack | meals | hungry | food | eat | meal |
| 3 | lbs | pounds | weight | lb | week | gained | exercise | pound |
